# Current News

**Published:** 1892-05

**Authors:** 


### Current News.

### DENTAL SOCIETY OF THE STATE OF NEW YORK.

THE Twenty-fourth Annual Meeting of the above Society will be held in the Y. M. C. A. Building, Albany, Wednesday and Thursday, May 11 and 12, commencing promptly at 10 o'clock, in the forenoon of the first day.

### ESSAYS TO BE READ.

"Dental Erosions and the Gouty Diathesis. Are they usually associated?" By Edwin T. Darby, D.D.S.

"Treatment of Irregularities of the Teeth." By Eugene S. Talbot, D.D.S.

"The Herbst Method of treating Exposed Pulps." By C. F. W. Bödecker, D.D.S.

"Electricity: Its Application in Dental Science." By Albert Carter Westlake, D.D.S.

The following distinguished members of the profession are to take part in the discussion, and will positively be in attendance:

Dr. A. C. Northrop, Dr. S. Y. Perry, Dr. E. C. Kirk, Dr. Daniel N. McQuillan, Dr. John D. Thomas, Dr. R. B. Winder, Dr. M. W. Foster, Dr. L. D. Shepard, Dr. J. N. Farrar, Dr. V. H. Jackson, Dr. Frank Abbott, Dr. L. Ashley Faught, Dr. W. C. Barrett, Dr. C. E. Francis, Dr. William H. Dwinelle, Dr. R. R. Andrews, Dr. Frank French, Dr. E. S. Gaylord, Dr. K. C. Gibson, Dr. C. N. Peirce,

Dr. S. H. Guilford, Dr. B. F. Luckey, Dr. F. H. Lee, Dr. Fred. A. Levy, Dr. W. W. Allport, Dr. S. C. G. Watkins, Dr. C. S. Stockton, Dr. E. A. Bogue, Dr. C. Heitzmann, Dr. W. G. A. Bonwill.

A. W. Walker, *President.*
Charles S. Butler, *Secretary.*

---

### AMERICAN DENTAL ASSOCIATION—SPECIAL NOTICE.

Those intending to present papers to the different sections of the Association, at the meeting to be held at Niagara, will aid very materially in the work if they will have duplicate type-written copies of the essays made. The labor and responsibility imposed upon the secretary of the Association by the demands of the various journals for abstracts has made this change a necessity, and it is especially urged that this request be complied with in all cases.

Signed by order of the Chairman of the Executive Committee.

C. N. Peirce,
*Secretary.*

---

### UNION MEETING OF THE PENNSYLVANIA AND NEW JERSEY STATE DENTAL SOCIETIES.

There will be a joint union meeting of the Pennsylvania and New Jersey State Dental Societies held at Cresson Springs, Pa., on July 20, 21, and 22. We expect this to be one of the largest dental meetings ever held in this part of the country.

All who wish to give clinics at this meeting, which will be one of unusual interest, can communicate during April and May with

P. K. Filbert, Pottsville, Pa.,
*Chairman of Clinic Committee for Pa. Society.*
S. C. G. Watkins, Montclair, N. J.,
*Chairman of Clinic Committee for N. J. Society.*

---

### ILLINOIS STATE DENTAL SOCIETY.

The Twenty-eighth Annual Meeting of the Illinois State Dental Society will be held at Springfield, Ill., May 10 to 13, 1892. The State Board of Dental Examiners meet at the same time and place. The profession generally is cordially invited.

Louis Ottofy,
*Secretary.*

70 Dearborn Street, Chicago.

### CHICAGO DENTAL SOCIETY.

At the annual meeting of the Chicago Dental Society, held Tuesday evening, April 5, 1892, the following officers were elected for the ensuing year:

J. W. Wassall, President; Thos. L. Gilmer, First Vice-President; E. A. Royce, Second Vice-President; L. L. Davis, Recording Secretary; Geo. J. Dennis, Corresponding Secretary; E. D. Swain, Treasurer; J. H. Smyzer, Librarian.

*Board of Directors.*—G. H. Cushing, E. Noyes, and J. G. Reid.

*Board of Censors.*—A. H. Peck, B. D. Wikoff, and D. M. Gallis.

Geo. J. Dennis,
*Corresponding Secretary.*

---

### PATENTS ISSUED.

A list of recent patents reported specially for the International Dental Journal:

470,184.—Dental Plugger. Edward Ebi, Cedar Rapids, Iowa. Filed October 25, 1890. Serial No. 369,290.

470,211.—Dental Mouth-Mirror. Robert F. Phillips, San Diego, Cal. Filed August 5, 1891. Serial No. 401,805.

470,332.—Artificial Tooth. Charles E. Friel, Boston, Mass. Assignor of one-half to Alfred H. Gilson, same place. Filed January 6, 1892. Serial No. 417,194.

470,670.—Dental Disk-Holder. F. F. Gilmore, Princeton, Ind. Assignor to the S. S. White Dental Manufacturing Company, Philadelphia, Pa. Filed July 1, 1891.

471,146.—Dental Engine. C. P. Shultz, New York, N. Y. Filed July 7, 1891.

---

### AMERICAN MEDICAL ASSOCIATION.

### SECTION OF ORAL AND DENTAL SURGERY.

The annual meeting of this Association will be held in Detroit, June 7, 8, 9, and 10.

The officers of the Section are: Professor J. Taft, Chairman; Eugene S. Talbot, Secretary.

The following essayists have promised to read papers:

Dr. J. Taft, Address; Dr. G. H. Gradle, Dr. G. S. Junkerman, Dr. John L. Gish, Dr. T. D. Crother, Dr. J. Smith Dodge, Dr. G.

Lenox Curtis, Dr. W. C. Barrett, Dr. M. H. Fletcher, Dr. E. S. Talbot, Dr. John S. Marshall, Dr. G. W. Weld.

EUGENE S. TALBOT,
*Secretary.*

125 STATE STREET, CHICAGO, ILL.

